# How to Integrate Sex and Gender Medicine into Medical and Allied Health Profession Undergraduate, Graduate, and Post-Graduate Education: Insights from a Rapid Systematic Literature Review and a Thematic Meta-Synthesis

**DOI:** 10.3390/jpm12040612

**Published:** 2022-04-11

**Authors:** Rola Khamisy-Farah, Nicola Luigi Bragazzi

**Affiliations:** 1 Clalit Health Services, Akko 2412001, Israel; rkhamisy@yahoo.com; 2 Azrieli Faculty of Medicine, Bar-Ilan University, Safed 13100, Israel; 3 Laboratory for Industrial and Applied Mathematics (LIAM), Department of Mathematics and Statistics, York University, Toronto, ON M3J 1P3, Canada

**Keywords:** sex- and gender-based medicine, medical education, medical training, curriculum building and improvement

## Abstract

Sex and gender are concepts that are often misunderstood and misused, being utilized in a biased, preconceived, interchangeable way. Sex and gender medicine is generally overlooked, despite the profound impact of sex and gender on health outcomes. The aims of the present rapid systematic literature review were (i) to assess the extent to which sex- and gender-sensitive topics are covered in medical courses; (ii) to assess the need for and willingness toward integrating/incorporating sex and gender medicine into health-related education; (iii) to identify barriers and facilitators of the process of implementation of sex and gender medicine in medical teaching, mentoring, and training; and (iv) to evaluate the effectiveness of interventional projects targeting curriculum building and improvement for future gender-sensitive physicians. Seven themes were identified by means of a thematic analysis, namely, (i) how much sex- and gender-based medicine is covered by medical courses and integrated into current medical curricula, (ii) the knowledge of sex and gender medicine among medical and allied health profession students, (iii) the need for and willingness toward acquiring sex- and gender-sensitive skills, (iv) how to integrate sex- and gender-based medicine into medical curricula in terms of barriers and facilitators, (v) existing platforms and tools to share knowledge related to sex and gender medicine, (vi) sex- and gender-based medicine aspects in the post-medical education, and (vii) the impact of sex- and gender-sensitive topics integrated into medical curricula. Based on the identified gaps in knowledge, further high-quality, randomized trials with larger samples are urgently warranted to fill these gaps in the field of implementation of gender medicine in educating and training future gender-sensitive physicians.

## 1. Introduction

“Sex” and “gender” are concepts that are often misused and misunderstood, being used in a preconceived, biased, and/or interchangeable way. Despite sounding like they overlap, they are two separate concepts. “Sex” is, indeed, a biological variable linked to the genetic/genomic (the so-called “chromosomal complement”) and post-genetic/post-genomic, endocrinological (the “hormonal complement”), and phenotypic/anatomic/phenomic (reproductive organs) components. In contrast, “gender,” which is a subjective variable (self-identification/self-declaration), relates to one’s personal, as well as societal, cultural, and political experiences. Both sex and gender variables and their subtle interactions can impact health-related outcomes [1]. 

The still-ongoing “Coronavirus Disease 2019” (COVID-19) pandemic showcases how profoundly clinical (diagnostic and prognostic) outcomes can be affected by sex and gender and how it is of crucial importance to consider such parameters [2,3].

Several healthcare organizations and institutions, like the USA “National Academy of Medicine,” previously known as the “Institute of Medicine” (IoM), and the Canadian Institutes of Health, have underlined how these variables are, indeed, not restrained to reproductive and sexual health and wellbeing only, but they concern the entire human organism as a whole, from a biological, as well as from psychological and societal points of view. Furthermore, devising strategies and conceptual frameworks that integrate sex- and gender-related aspects would influence the way healthcare provisions are delivered and, generally speaking, all the biomedical practices. This would pave the way for the achievement and implementation of the so-called personalized/individualized medicine, where the precise needs of each individual are taken into account, instead of a “one-size-fits-all” approach.

Sex- and gender-specific differences can, indeed, dramatically impact the natural story of a disease, from its etiopathogenesis to the response to treatment [4].

Despite the importance of this topic, sex- and gender-specific medicine is still overlooked in the academic arena [4], with information related to sex- and gender-related biomedical aspects being neglected or inconsistently incorporated/integrated into the medical and allied health profession undergraduate, graduate, and post-graduate training and curricula.

Concerning COVID-19, research has found that, despite the implications of sex and gender for COVID-19 diagnosis, treatment, and prognosis, these variables are rarely accounted for. For instance, out of 75 COVID-19 vaccines trials, only 24% explicitly presented endpoint outcomes (i.e., vaccine effectiveness and safety profile) stratified by sex and only 13% of them discussed sex-specific differences [5].

All this shows the importance of integrating/incorporating sex- and gender-based medicine into clinical teaching. Sex- and gender-based medicine has been classically defined as “a study of the differences in men’s and women’s normal function and in their experience of the same diseases,” according to Marianne Legato [6].

The aims of the present rapid systematic literature review were (i) to assess the extent to which sex- and gender-sensitive topics are covered in medical courses; (ii) to assess the need for and willingness toward integrating/incorporating sex and gender medicine into health-related education; (iii) to identify barriers and facilitators of the process of implementation of sex and gender medicine in medical teaching, mentoring, and training; and (iv) to evaluate the effectiveness of interventional projects targeting curriculum building and improvement for future gender-sensitive physicians.

## 2. Material and Methods

### 2.1. Review Study Protocol

The review study protocol was a priori devised according to the “Preferred Reporting Items for Systematic Reviews and Meta-Analyses-Protocol” (PRISMA-P) 2015 guidelines [7]. The protocol is registered within the Open Science Framework (OSF) registry (ID 10.17605/OSF.IO/3XQ4R).

### 2.2. Literature Search

A rapid systematic review of the literature [8] was conducted by mining PubMed/MEDLINE (The National Library of Medicine, The National Institutes of Health, Bethesda, MD, USA), the major electronic database of the scholarly biomedical literature, searching for the following keywords: “sex and gender medicine,” “sex and gender-based medicine,” “gender medicine,” “sex and gender health,” “sex,” “gender,” “gender-sensitive medicine,” “medical training,” “medical mentoring,” “medical education,” “medical syllabus,” “nursing training,” “nursing mentoring,” “nursing education,” “future doctors,” “future practitioners,” and “future physicians.” Wild-card option and Medical Subject Headings (MeSH) terms were utilized when necessary. Proper Boolean connectors were utilized. The search string was purposely broad in order to curb the chance of missing potentially relevant articles.

One database was deemed enough since a rapid systematic review of the literature generally includes a streamlined methodology with respect to classic approaches deployed in conducting systematic reviews of the literature (including mining only one database).

### 2.3. Inclusion and Exclusion Criteria

Inclusion and exclusion criteria were devised according to the population/intervention (exposure)/comparator (comparison)/outcome/study design (PICOS) criteria. Inclusion criteria were original studies of any type in which either qualitative/quantitative or mixed methods research had been conducted (S), surveying medical or allied health profession students (at the undergraduate, graduate, or postgraduate level), as well as professors from medical faculties (P).

Articles were retained if performing any comparison, including attendance of sex- and gender-sensitive seminars, lectures, or workshops versus nonattendance; assessing the best form of delivering gender-sensitive topics (modular versus mainstream or no course of sex and gender medicine; lecture versus seminar or workshop; or investigating the impact of the gender of the attendant (male versus female), among others).

Articles dedicated to the implementation of “lesbian, gay, bisexual, transexual/transgender” (LGBT)/“lesbian, gay, bisexual, transexual/transgender/queer/intersex” (LGBTQI)-related healthcare were excluded from the present review. Studies assessing the exposure of students to men’s health, women’s health, gender diversity, gender inclusion, and related topics were not retained in the present review. Articles surveying students and/or professors from non-medical faculties were excluded as well.

Further details are reported in Table 1, to which the reader is referred.

Extensive cross-referencing was applied, manually screening the reference list of each potentially eligible article. Target journals, including *Biology of Sex Differences*, *Health Care for Women International*, *Journal of Women’s Health (Larchmt)*, *GMS Journal of Medical Education*, *BMC Medical Education*, *Gender Medicine*, and *Education for Health (Abingdon)*, were hand-searched.

### 2.4. Data Abstraction and Synthesis

Relevant data were abstracted by two authors independently (R.K.-F., N.L.B.) from retained studies and were synthesized using a thematic approach [9]. In the case of potential disagreement, a consensus was reached through discussion.

### 2.5. Critical Quality Appraisal and Risk of Bias Assessment

Critical quality appraisal and risk of bias were not performed since they are streamlined steps within the rapid systematic review literature methodology [8].

### 2.6. Findings Reporting

The findings of the present rapid systematic review are reported according to the “Preferred Reporting Items for Systematic Reviews and Meta-Analyses” (PRISMA) 2020 guidelines [10].

## 3. Results

The initial literature search yielded a pool of 8464 items. After removing duplicates and discarding irrelevant articles, twenty-five studies [11,12,13,14,15,16,17,18,19,20,21,22,23,24,25,26,27,28,29,30,31,32,33,34,35,36,37,38] were retained in the present literature review. Seven major themes could be identified, namely, (i) how much sex and gender medicine are covered by medical/allied health profession courses and integrated/incorporated into current medical curricula, (ii) the knowledge of sex and gender medicine among medical and allied health profession students, (iii) the need for and willingness toward acquiring sex- and gender-sensitive skills, (iv) how to integrate/incorporate gender medicine into medical curricula in terms of barriers and facilitators, (v) existing platforms and tools to share knowledge related to sex and gender medicine, (vi) sex and gender medicine aspects in the post-medical education, and (vii) the impact of sex- and gender-sensitive topics that are integrated/incorporated into medical curricula.

### 3.1. How Much Sex and Gender Medicine Is Covered by Medical Courses and Integrated into Current Medical Curricula

Sex- and gender-based medicine is rarely and inconsistently covered by medical courses and is not organically/coherently integrated/combined into medical curricula. Thande et al. [11] assessed a medical curriculum in terms of sex- and gender-based content. More specifically, first- and second-year medical students from the Yale School of Medicine, USA, attended 548 lectures and workshops and less than 25% of these sessions spoke about the impact of sex and gender variables on human health and disease and the effects on patient care, with only 8% of them discussing the influence of sex and gender on human physiology and pathophysiology. Moreover, when discussed and introduced, these topics were disseminated via lectures rather than during small group workshops, which are more useful for acquiring critical clinical reasoning competence and skills. Furthermore, sex- or gender-related topics were generally discussed and presented in binary terms, rather than as a continuum, with a focus on underlying biological processes and phenomena, almost completely overlooking psycho-social aspects.

Miller et al. [12] carried out a sex- and gender-based medicine survey, recruiting 44 medical schools from the US and Canada. Most of them (70%) stated that they did not have a formal sex- and gender-specific integrated/combined medical curriculum. Coverage of sex- and gender-specific differences for ten health-related topics was judged as minimal from 45% to 70% of respondents.

In another study, Miller et al. [13] obtained similar results. The authors assessed sex- and gender-based medical knowledge in a sample of 72 US medical students from the Mayo Medical School, who were administered a 35-item questionnaire. More than half of the participants indicated that sex- and gender-sensitive topics were covered in courses, including gynecology, pediatrics, and cardiology. A total of 42% and 30% of the respondents stated that these topics were addressed in oncology and gastroenterology, respectively, with less than 20% of the students stating that those topics were included in other courses, such as neurology, orthopedics, nephrology, or immunology.

Henrich and Viscoli [14] carried out an analysis of the curriculum of 95 US medical schools to see how many of them offered gender-specific courses/clerkships. Twenty-four gender-specific topics could be identified, with fewer than 30% of the medical schools included in the analysis providing gender-specific topics.

In another study, Henrich et al. [15] surveyed a sample of 1267 third- and fourth-year students from 101 US allopathic medical schools, who indicated brief-to-moderate coverage of sex- and gender-sensitive topics and moderate preparedness for gender-sensitive skills. After a multivariate regression analysis, being female was associated with lower perceived preparedness.

Finally, Nocon et al. [16] surveyed a random sample of 136 participants, including university professors and assistant medical directors specializing in different medical specializations (namely, gynecology, cardiology, and neurology) at German university hospitals. Most respondents (83%) judged the current importance of sex and gender medicine in Germany as low, with 62% of them stating that it should be a required topic during medical studies due to it being highly useful in the improvement of diagnosis (72%), delivery of healthcare provisions and treatment (80%), avoidance of complications (77%), and reduction in mortality (64%) and generated costs (73%).

### 3.2. The Knowledge of Sex and Gender Medicine among Medical and Allied Health Profession Students

In the study by Miller et al. [13], the median number of students providing correct answers was 20 (52.6%, ranging from 0 to 37) for the fourth year of medical school and 17 (51.5%, ranging from 1 to 32) for the second year. Approximately 75% of the respondents in both years were aware of the difference between sex and gender, even though only a few of them (*n* = 4 from the second year) correctly stated that clinical trials should provide outcomes stratified by gender.

Bisconti et al. [17] evaluated the need and willingness of a sample of 617 Italian physiotherapists to deepen their knowledge in the field of sex and gender medicine. The participants were administered an 18-item questionnaire: most of them (68%) had only a general knowledge of sex and gender medicine, with 55% of the respondents reporting a low-to-moderate understanding/knowledge of it. Knowledge related to sex- and gender-based medicine did not differ between male and female respondents. On the other hand, more than 92% of the recruited sample perceived the importance of the topic and believed they should receive ad hoc training in the field. After a multivariate regression analysis, being a male (odds ratio (OR) of 1.43 [95% confidence interval (CI) 1.01–2.05], *p* = 0.04) and working in a private entity (OR 2.36 [95%CI 1.51–3.69], *p* = 0.00) was associated with applying the principles of sex- and gender-based medicine in the daily practice of physiotherapy. Other statistically significant predictors were knowledge/understanding of sex- and gender-sensitive topics, their perceived importance and impact on human health and disease, and need/willingness to attend courses/lectures/workshops or clerkships related to sex- and gender-based medicine.

### 3.3. The Need for and Willingness toward Acquiring Sex- and Gender-Sensitive Skills

Exenberger et al. [18] evaluated the academic courses taught at the Medical University of Innsbruck, Austria, and surveyed a vast sample (*n* = 536) of instructors, lecturers, and educators about the teaching and testing content. The respondents felt that communicative and social competencies and skills were of crucial importance in the field of medical education, teaching, and training.

Steinböck et al. [19] described the implementation of sex and gender medical-related elective subjects at the Medical Universities of Innsbruck and Vienna, Austria. The two-lecture series were comparable in terms of topics taught and material utilized and were well-received by students.

Scholte et al. [20] conducted an explorative thematic document analysis of educational assignments containing sex- and gender-sensitive components made by successful aspiring medical students (*n* = 50). Applicants were willing to acquire skills to become gender-sensitive doctors, strongly advocating for inclusive healthcare and equity in access to health provisions. According to the respondents, the best path toward fully incorporating sex and gender medicine into daily clinical practice should start from acquiring basic biomedical knowledge about sex- and gender-specific differences and their impact on human health and disease, gradually developing social and communicative skills, and competence. The deployment of audiovisual interactive materials and simulated practical situations could be useful within this strategy. Finally, students emphasized the importance of having gender-sensitive mentors and educators during medical studies.

Gaida et al. [21] described the design and implementation of the elective gender-sensitive subject “career management for medical students” taught at Leipzig University, Germany, since 2010/2011. According to a survey, approximately one-third of the medical students of the university were willing to study gender-sensitive topics.

Jenkins et al. [22] surveyed a sample of 1097 US allopathic- and osteopathic-enrolled students about their attitudes toward sex and gender medicine. The majority of the participants were convinced of the importance and impact of a sex- and gender-sensitive approach on healthcare (96%) and thought that sex and gender medicine should be an important component of medical curricula (94%), with only slightly more than 2% of the respondents thinking that sex and gender medicine was equivalent to women’s health or medicine. From 19% to 41% of the participants were unaware of the incorporation/integration of sex- and gender-sensitive topics into their curricula, with males reporting a higher rate of exposure to sex and gender medicine than their female counterparts. Moreover, only 35% of the participants reported that they would be prepared to manage sex- and gender-specific differences in healthcare.

van Leerdam et al. [23] performed a focus group study related to the exposure to gender-sensitive topics during internal medicine or surgical clerkships, surveying a sample of 29 medical students from Radboud University, Nijmegen, the Netherlands. The respondents stated that they had rarely been exposed to such topics, mainly due to insufficient knowledge, lack of awareness, and perceived minor impact or irrelevance of sex and gender medicine. Medical students were, however, willing to deepen their knowledge and acquire sufficient competencies and skills within the field of sex and gender medicine.

Finally, Böckers et al. [24] described the design and implementation process of a lecture-based, multidisciplinary, longitudinal, basic sex- and gender-sensitive curriculum consisting of 15 lecture sessions throughout the five years of the medical school. The project took place at Ulm University, Germany. The authors also evaluated students’ knowledge and attitudes related to sex and gender medicine. The curriculum was highly accepted (by more than 80% of medical students). However, there were aspects of rejection and/or resistance to the introduction and implementation of sex and gender medicine, with approximately half of them judging the material as informative or relevant to their clinical daily practice as doctors. Interestingly, students from the latest years and males were less willing to accept the newly designed curriculum.

### 3.4. How to Integrate Gender Medicine into Medical Curricula: Barriers and Facilitators

Verdonk et al. [25] qualitatively investigated the changes implemented to a gender-related biomedical program devised since 1998 at the Radboud University Nijmegen Medical Centre, the Netherlands. The authors screened the syllabus and educational material of nine curricular blocks in terms of language, context, and content, and made significant amendments and improvements to make it more inclusive, integrating gender-related issues. Then, the authors conducted in-depth interviews with block coordinators and found that various factors had played a key role in favoring and facilitating the incorporation of gender-medicine-related aspects into the biomedical curricula. The presence of a “trigger person” and the motivation of the block coordinators, together with their engagement and involvement in every step of the decision-making processes, the provision of concrete support, pragmatic proposals concerning curricular adjustments in a content-oriented fashion within an already well-established and consolidated program, and showing how sex- and gender-specific differences deeply impact healthcare delivery to the patient, were the major factors leading to the success of the curricular incorporation of gender medicine into medical syllabuses.

Park et al. [26] developed a graduate course at Seoul National University College of Medicine, South Korea, named “Sex and gender aspects in biomedical research” and surveyed a sample of twelve students from medicine, community nursing/nursing system, and public health, along with ten professors. The material taught included textbooks, online courses, and scholarly articles. Respondents stated that sex- and gender-sensitive governmental policies and the allocation of dedicated funding were key factors, playing a major role in the implementation of sex and gender medicine.

Ludwig et al. [27] devised an innovative, modular, outcome-based, multidisciplinary undergraduate curriculum integrating sex- and gender-sensitive topics. The introduction and involvement of a “gender change agent” into the curriculum development team facilitated the development of the new, modular curriculum. All relevant stakeholders were engaged in a 10-step systematic approach aimed at identifying, selecting, and introducing/placing sex- and gender-related topics, as well as advising faculty members and monitoring the outcomes and effectiveness of the integration/incorporation process. The newly developed curriculum consisted of 94 lectures, 33 seminars, and 16 courses with a practical focus. Sex- and gender-sensitive topics represented 5% of the learning objectives. Biological, psychological, and socio-cultural aspects were coherently integrated into the curriculum.

The 10-step approach implemented by Ludwig et al. [27] was similar to the eight-step approach proposed by Chin et al. [28], namely, (i) to communicate a sense of urgency related to the need of implementing sex and gender medicine as extremely impacting on patient healthcare and on the goals of individualized/personalized medicine; (ii) to build a strong team involving stakeholders (including well-known experts, researchers and other scholars, mentors/educators/instructors/teachers/lecturers, as well as learners, students, and patients); (iii) to develop a clear vision and mission, exploiting extant tools and resources; (iv) to communicate, share, and disseminate such as vision; (v) to empower people (for instance, students and learners); (vi) to develop concrete, short-term goals, and content-oriented proposals; (vii) to consolidate and expand the previous processes; and (viii) to “institutionalize the change” by devising a list of fundamental sex and gender medical-related competencies and skills.

Moreover, Chin et al. [28] and McGregor et al. [29] suggested capitalizing on successful experiences of curricular integration and combination (women’s health, emergency medicine, and LGBT/LGBTQI health and wellbeing).

In a study by Nachtschatt et al. [30], it was suggested that, after being integrated into the biomedical curriculum, the real challenge was to ensure implementation in a sustainable way.

Finally, building on previous research and proposals, Tannenbaum and Moineau [31] suggested adopting a system-level approach: combining a top-down (accreditation pressure) strategy with a bottom-up (empowering students as catalysts of change and innovation) approach.

### 3.5. Existing Platforms and Tools to Share Knowledge related to Sex and Gender Medicine

Auditing and mapping extant sex- and gender-sensitive topics is a good practice, as indicated by Song et al. [32]. Existing platforms and tools that were devised ad hoc to share and disseminate knowledge regarding sex and gender medicine are overviewed in Table 2.

For instance, Seeland et al. [33] described the design and implementation of an e-learning and knowledge-sharing platform that was specifically developed for sex and gender medicine. This open-source, user-friendly, web-based tool was termed the eGender platform and can be accessed at http://egender.charite.de, accessed on 2 February 2022. The platform was devised with the aim of providing future health professionals with adequate knowledge and communication competence and skills on sex- and gender-specific differences and their impact on human health and disease. From a pedagogic and educational perspective, the tool can support a blended learning teaching framework and is based on the concept of constructivism. The content is of high quality and has been specifically developed by experts from seven universities: it is both of broad scope and nature and specific to each medical specialization. Modules consist of textual, audio-visual, and interactive material, and the number of registered users has been gradually increasing, showing more interest in sex and gender medicine.

“GenderMed-Wiki” (accessible at www.gendermed-wiki.de, accessed on 2 February 2022) is an online exchange platform funded by the German Federal Ministry of Education and Research [34]. Schreitmüller et al. [34] qualitatively evaluated this tool by means of four focus groups (totaling thirty participants, consisting of students, lecturers/instructors/mentors, physicians, and the lay public). Feedback obtained from focus groups resulted in improvements and optimization of the tool concerning four major aspects, namely, (i) the content, (ii) technical issues and requirements of the platform, (iii) its usability, and (iv) legal and ethical aspects. Subsequently, a quantitative study was conducted, interviewing 149 medical and dentistry students from the Universities of Muenster and Duisburg-Essen. Even though it was deemed of high quality and edifying and informative, the tool was not judged relevant and pertinent to the medical and dentistry curricular studies. However, participants thought the tool could have been useful during clinical practice. Interestingly, judgments regarding relevance/pertinence correlated with lower knowledge levels about sex and gender medicine.

A PubMed research tool specifically built for retrieving sex- and gender-related biomedical scholarly literature was described in the study by Song et al. [32].

The GenderMed database (http://gendermeddb.charite.de, accessed on 2 February 2022) is developed by the Institute of Gender in Medicine (GiM), based at the Charite University Hospital, Berlin, Germany. The FUTURE platform includes the open knowledge database developed by Stanford University and adjusted for the Swedish audience (accessible at www.genderedinnovations.se, accessed on 2 February 2022) [35].

Another existing tool is the Canadian “Gender Lens Tool.” Weyers et al. [36] translated and adapted it into German. The cultural adjustment was validated by five teachers and four students. After the reliability and quality assessment, it was administered to a cohort of 247 fourth-semester students during a seminar related to sex and gender medicine, receiving further feedback.

Finally, the Online Continuing Medical Education and Certificate Program in Sex- and Gender-Specific Health, under development with the support of the Laura W. Bush Institute for Women’s Health, is a continuing professional development site (http://www.laurabushinstitute.org/cme/default.aspx, accessed on 2 February 2022).

### 3.6. Sex and Gender Medicine Aspects in Post-Medical Education

Kling et al. [37] performed a cross-sectional, questionnaire-based study among 271 residents at the Mayo Clinic and analyzed the data by carrying out descriptive and qualitative thematic analyses. More than 70% of the interviewees said that they had never (16%) or occasionally (55%) had the opportunity to discuss with their instructor/preceptor/mentor about the impact of sex and/or gender on patient healthcare. Approximately half of the respondents (48%) were unsure and/or unaware of sex- and gender-specific differences, not fully understanding the impact of such variables on human health and disease and/or believing that these parameters were unrelated to their medical specialization. On the other hand, female residents were aware of the importance of sex and gender medicine (60% vs. 39%), even though more male residents had taken part in sex- and gender-related biomedical research than their female counterparts (60% vs. 39%).

Dhawan et al. [38] performed a survey among 80 medical residents and fellows at Cedars-Sinai Medical Center, USA, about their awareness of sex and gender medicine. Approximately seventy percent of the respondents replied that sex- and gender-related concepts had been never or very rarely introduced during their training programs. On the other hand, slightly more than 60–65% of the participants felt that these concepts were important and should be incorporated into clinical residency.

Finally, Dielissen et al. [39] evaluated the impact of teaching sex- and gender-medicine-related aspects on the training of general practitioners, performing a prospective study in the Netherlands. Trainees were exposed to a modular (*n* = 75) or mainstream (*n* = 72) gender medicine program. A third cohort of general practitioner trainees (*n* = 60) acted as the control cohort. Participants were administered the “Nijmegen Gender Awareness in Medicine Scale” and a 16-item questionnaire concerning gender knowledge. The modular approach toward teaching gender medicine was found to statistically significantly improve gender knowledge (*p* = 0.049), whereas the three cohorts did not differ in terms of gender sensitivity or gender role ideology. Interestingly, female GP trainees were more gender-aware than their male counterparts.

### 3.7. The Impact of Sex- and Gender-Sensitive Topics Integrated into Medical Curricula

Integrating sex- and gender-sensitive topics into medical curricula significantly improved students’ knowledge of sex and gender medicine. Siller et al. [40] surveyed a sample of 483 students (352 females, 131 males) from the faculty of medicine, as well as allied health professions, on the impact of attending sex and gender medicine lectures and workshops on awareness and attitudes toward sex- and gender-sensitive topics. The authors were able to find a positive association between lectures attendance and knowledge/attitudes. Interestingly, male participants benefited from lectures more than their female counterparts.

In a study by Chin et al. [28], the outcomes of attending an educational summit on sex- and gender-based medicine were evaluated in a sample of 148 attendees. Knowledge of sex- and gender-sensitive topics increased from 81% to 93%. A total of 69% of the participants thought that drug dosage should be considered according to the sex of the patient before attending the summit and increased up to 97% after the summit. Moreover, 40% of the respondents believed sex- and gender-based medicine was an instrumental component of precision medicine: this percentage increased up to 81% after attending the summit. Most of the participants had changed their opinion concerning the importance of sex- and gender-based medicine (61% yes and 22% somewhat), whereas only 17% had not changed their mind.

In a study by Park et al. [26], participants (both professors and students) were administered a four-item questionnaire; the results showed that they were unfamiliar with sex- and gender-specific differences and their impact on human health and disease and with the concept of “gendered innovation.” The course was useful to expose them to these new conceptual frameworks, which they realized had great importance in biomedicine, both in clinical practice and research. Interestingly, there were no differences in terms of sex- and gender-specific differences between students and professors, or between males and females.

## 4. Discussion and Future Prospects

Addressing healthcare disparities and inequities and ensuring equitable and customized access to healthcare provisions are a societal onus and a crucial step for implementing initiatives and programs based on precision and individualized/personalized/stratified medicine. Sex and gender medicine should be a fundamental component of medical curricula, being a major asset of precision medicine. Incorporating and integrating sex- and gender-related variables into clinical research and practice would significantly enhance and deepen our understanding of the subtle, complex, and non-linear impact of sex and gender on human health and disease.

However, findings from the present rapid systematic review of the literature show that the majority of medical students, as well as clinical residents and fellows, have never had or rarely had the opportunity of discussing the topic of sex and gender medicine with their instructors, even though they perceive the importance of such topics. Current medical curricula, both at the graduate and post-graduate levels, suffer from profound gaps with regard to the implementation of teaching components related to sex and gender medicine.

Moreover, there are barriers to the full integration of sex- and gender-specific knowledge into the biomedical syllabus, which include preconceived, biased opinions and ideas about sex and gender, the lack of adequate learning material and systematic teacher training, and an innovative strategy based on social and communicative competence and skills, as well as a perceived minor impact, translational added value, or even irrelevance of sex and gender medicine on daily clinical practice [41]. Furthermore, curricular reforms and shifts are difficult to implement, given their complexity, with faculty members and administrators being overburdened and overloaded. On the other hand, the identification and involvement of a “gender change agent”; the early engagement, motivation, and commitment of all relevant stakeholders; and the allocation of dedicated funding can facilitate relevant changes. Barriers and facilitators to the incorporation/integration of sex and gender medicine into academic teaching and clinical practice are overviewed in Table 3.

Based on the findings of the articles overviewed in the present review, we can identify the main desired features of a sex and gender medical course. This should be longitudinally integrated with other (basic and applied/clinical/translational) courses, with a strong clinical focus, covering multiple competency levels and making use of web/interactive resources and tools, with measurable learning objectives and outcomes and concrete, content-oriented, practical goals. These and other desired features of a sex- and gender-medicine course are overviewed in Table 4.

Equipping future physicians with a clear understanding of the influence and effects of sex and gender on health is paramount in advancing the achievements of patient-centered care.

It is, therefore, crucial to change the attitudes of medical students toward sex-/gender-sensitive medicine.

To the best of our knowledge, this is the first systematic review on the topic of integrating sex and gender medicine into medical curricula. Our review significantly complements and adds to the narrative reviews by Ruiz-Cantero et al. [42] and by Lagro-Janssen [43], and the scoping review by Siller et al. [44].

## 5. Conclusions

COVID-19 as a showcase of the so-called “gendered diseases” has highlighted the importance of integrating and incorporating sex- and gender-sensitive topics into the biomedical curriculum. The present rapid systematic review of the literature identified seven main educational themes, including the major barriers and facilitators of the implementation of sex and gender medicine in the educational training of medical students, residents, and fellows [45]. However, based on the identified gaps in knowledge, further high-quality, randomized trials with larger samples are urgently warranted to fill these gaps in the field of implementation of sex and gender medicine in educating and training future sex- and gender-sensitive physicians. Moreover, since practically all the studies included in the present rapid systematic review of the literature were based in North America and Europe, with a few exceptions (such as the study in South Korea), future investigations should be carried out in other countries and settings.

## Figures and Tables

**Table 1:** Inclusion and exclusion criteria adopted in the present rapid systematic review of the literature.

PICOS Component	Inclusion Criteria	Exclusion Criteria
P (population)	Medical or allied health profession students (at the undergraduate, graduate, or postgraduate levels), including clinical residents, and fellows Professors/lecturers/educators from medical faculties and schools	Students (at the undergraduate, graduate, or postgraduate level) attending other non-medical faculties Professors/lecturers/educators from other non-medical faculties
I (intervention/exposure)	Exposure to sex- and gender-sensitive topics	Exposure to LGBT/LGBTI-related topics Exposure to men’s health and related topics Exposure to woman’s health and related topic Exposure to gender-diversity- and gender-inclusion-related topics
C (comparator/comparison)	Any comparison, including attendance of sex- and gender-sensitive seminars, lectures, or workshops versus nonattendance; modular versus mainstream or no course of sex and gender medicine; lecture versus seminar or workshop; impact of the gender of the attendant (male versus female)	Any other comparison
O (outcomes)	Change in sex and gender medicine knowledge, acceptance of sex and gender medicine from students and professors/lecturers/educators	Any other outcomes
S (study design)	Original study (of any type, qualitative/quantitative/mixed methods, questionnaire/survey-based or focus-group-based), conference proceeding, education summits, commentaries, editorials with sufficient informative details	Reviews (of any type), editorials, commentaries without sufficient informative details

**Table 2:** Available tools/platforms/online courses for sharing and disseminating knowledge related to sex- and gender-based medicine.

Tool/Platform/Online Course	Website URL
EGender	http://egender.charite.de, accessed on 2 February 2022
GenderMed-Wiki	www.gendermed-wiki.de, accessed on 2 February 2022
GenderMed	http://gendermeddb.charite.de, accessed on 2 February 2022
The Online Continuing Medical Education and Certificate Program in Sex- and Gender-Specific Health	http://www.laurabushinstitute.org/cme/default.aspx, accessed on 2 February 2022
Sex and Gender Women’s Health Collaborative	http://www.sgwhc.org, accessed on 2 February 2022
The Texas Tech University Health Sciences Center PubMed Search Tool and Slide Library	https://ttuhsc.libguides.com/pubmed1, accessed on 2 February 2022
The Stanford University Center for Gendered Innovation, the European Union Research and Innovation, and the National Science Foundation	www.genderedinnovations.eu, accessed on 2 February 2022
The Science of Sex and Gender in Human Health offered by the Office of Women’s Health, National Institutes of Health, Office of Women’s Health, and the US Food and Drug Administration	http://sexandgendercourse.od.nih.gov, accessed on 2 February 2022
The Gender and Health Collaborative Curriculum Project with six collaborating medical schools of the Council of Ontario Faculties of Medicine, Canada	http://www.genderandhealth.ca/, accessed on 2 February 2022

**Table 3:** Barriers and facilitators of the implementation of sex and gender medicine in biomedical curricula.

Barriers
Preconceived, biased opinions and ideas about sex and gender
Insufficient knowledge related to sex and gender medicine among instructors/mentors/lecturers/professors
Unawareness of sex and gender medicine among instructors/mentors/lecturers/professors
Perceived minor impact, translational value, or irrelevance of sex and gender medicine on daily clinical practice
Lack of adequate, high-quality teaching material
Lack of a centralized repository for sex- and gender-related medical materials
Limited curricular space for integrating sex- and gender-based medicine
Complexity of curricular reforms and shifts
Accreditation procedures and related processes that could delay the implementation of integrating sex- and gender-based medicine
Overburdened faculty members and administrators
Once sex- and gender-based medicine has been integrated, implementation should be sought in a sustainable way
**Facilitators**
Involvement of a “gender change agent”/“trigger person”/“faculty champion”
Motivation of block coordinators and all members of the curriculum improvement team
Development and establishment of an ad hoc advisory committee consisting of medical school curriculum experts to oversee the entire process of implementation of a sex- and gender-based medical curriculum
Early involvement of all relevant stakeholders adopting a system-level approach: top-down (accreditation pressure) and bottom-up (empowering students as catalysts of change and innovation)
Faculty development
Support from faculty members
Governmental/institutional policies and mandate
Pragmatic proposals concerning curricular adjustments in a content-oriented fashion within an already well-established and consolidated study program
Allocation of dedicated funding
Time investment and commitment toward implementing a sex- and gender-based medical curriculum

**Table 4:** List of desired/required features of a potentially successful sex- and gender-based medical curriculum.

Desired/Required Features
Longitudinally integrated (“thread”) with other courses during medical studies
Integrated within both basic and applied/clinical/translational courses
Emphasizing the clinical meaning of incorporating sex- and gender-specific differences
Exploiting all currently available tools and resources
Encompassing all health, physiological and pathophysiological conditions
Teaching students how to adopt a “gender lens” so that they are able to identify and overcome gender biases, gaps, and inequities/disparities
Measurable learning objectives and concrete, content-oriented and practical goals
Ongoing monitoring phases
Multiple competency levels
Assessing student’s competency according to Miller’s pyramid (“knows, knows how, shows how, and does”)
Being content-oriented, problem-based, symptom-based, and goal-based

## Data Availability

The present article is a review, based on already published and available data.
